# Royal Westminster Ophthalmic Hospital

**Published:** 1829-10-01

**Authors:** 


### ROYAL WESTMINSTER OPHTHALMIC HOSPITAL.

Several cases for operation of various kinds have occurred in this hospital during the last month: three for entropium; two for the removal of the anterior part of the eye-ball; five for cataract, and one for artificial pupil.

The following for congenital cataract is the most deserving of attention, as the case had been considered irremediable; indeed Mr. Guthrie in deciding on an operation declared, that he did so in order to give the child every chance that surgery afforded, satisfied that no evil would ensue if the operations should not succeed. The result has, however, been successful, and very gratifying to all who have witnessed the difference in the appearance of the child since it has been made to see. At first it had but an indistinct idea of the form or distances of things, but it is rapidly acquiring that knowledge which is only to be obtained by the sense of feeling and by time.

Mary Check, aged four and a half years, living at 52, Praed Street, Paddington, was admitted April 4th, 1829. The child was blind, and had been so from her birth, being scarcely able to distinguish when it was daylight. There was a cataract in the right eye with the iris adhering to it; the pupil nearly closed, and only dilatable in a trifling degree by the belladonna. In the left eye there was also cataract with adhesions of the iris, the pupil was, however, more regular, and could be considerably dilated. The iris in both eyes looks rough and irregular, as if it had been the subject of inflammation. The mother says she has shown it to several gentlemen, who said there was nothing to be done for its relief.

May 28th. The pupils being dilated as much as possible, the following operations were performed. An artificial pupil was first made in the right eye by dividing the iris in the centre; the lens and capsule were then separated as much as possible from their adhesions to the iris, and broken up. The cataract in the left eye, after being detached from the iris, was depressed. A drop of the solution of belladonna was applied to each eye immediately after the operation, and this has been continued twice a day since. The pieces of lens are now rapidly dissolving, and the child begins to see very well. The inflammation which followed the operation was very trifling, and was completely reduced by cupping on the left temple to four ounces.

In the following case the existence of cataract in both eyes was not doubted, and the woman was sent up from the North of Scotland to undergo the operation.

Ann Barclay, aged 39, admitted April 23d, 1829, being almost blind. Says that she has lost the sight of the right eye for a twelve-month, and that for a month previously she suffered from pains in the head and a sense of heaviness over the brow, for which she was bled without any relief; blindness ensued a fortnight after she first perceived any impairment of vision. Three months back the left eye began to be affected similarly to the right; there was a cloudy appearance with black spots continually before her which prevented her seeing objects distinctly, even at a very short distance. This dimness has at the present time increased so as to cause blindness. She now complains of severe pains over her brow, and in her head; menstruation had been obstructed two years before the right eye was attacked, and is now very irregular; the lenses of both eyes appear muddy, and some gentlemen even thought that cataract existed; the iris acts freely but does not look very healthy. She was bled to 16 ounces, and ordered to take two pills composed of equal parts of blue pill and gamboge.

May 4th. Thinks she sees rather better; still complains of a sense of weight over the brow.

Cucur. C. ad ℥viij.

7th. Sees much better with the left eye, and can distinguish light from darkness with the left; pain in the head much relieved.

Cucur. C. ad ℥viij.
Rep. Pil. Hyd. cum Camb.

Since then she has been cupped five times; ten or twelve ounces of blood having been taken each time; blisters have been frequently applied behind her ears, and to the nape of her neck, and she has continued to take the pills regularly. She can now see to walk by herself tolerably well, and to distinguish persons accurately if the light is not too strong; the pains in the head and brow return occasionally:—to continue the pills.

XL.

**ROYAL WESTMINSTER OPHTHALMIC HOSPITAL.**

Mr. Guthrie has been lately giving a trial to the oleum terebinthinæ in cases of inflammation of the iris and choroid coat, on the plan recommended by Mr. Hugh Carmichael, of Dublin, and with a nearly analogous result. In some cases it has succeeded admirably; in others it has been of little service; and in some, unequal to the cure of the complaint. The irritation produced on the bladder and kidneys, is occasionally severe, after the exhibition of a very small quantity, whilst, much larger doses are borne by others with a perfect immunity from it. We have selected six cases illustrative of these points, and only premise that the turpentine has always been given in a solution of gum Arabic and nitre, and the trial has been made in common, rheumatic, or arthritic, and syphilitic inflammations. Mr. Guthrie thinks the remedy most valuable in the arthritic inflammations, which do not yield to mercury alone, but seem to be amenable to the conjoined effects of it and the turpentine, or to the latter alone.

*Case* 1. Mary Tyler, æt. 16, admitted July 3d, 1829, having iritis of the right eye, which has been inflamed for the last week. Says that she has not long recovered from an attack of inflammation of the same kind in the left, for which she took mercury. The pupil is irregular, and the colour of the iris changed; the conjunctiva and sclerotica are not much affected; the vessels of the latter form a zone of a pink color round the cornea; the discharge of tears and intolerance of light are very trifling and the cornea is quite transparent; her sight is however very much impaired. She complains principally of the pain over her brow which becomes very severe when she is in bed, so much so, that she has had very little rest since the commencement of the inflammation. There is a lichenous eruption on her body looking very much like the syphilitic, but she denies ever having had that disease, and says, that the eruption appeared about four months back, immediately after her recovery from the small pox.

Capiat Ol. Terebinth. ʒi. ter die.

July 4th. The medicine has produced a very decided and beneficial effect; the pupil is a little more dilated and quite circular, and she suffered much less pain than usual last night; the inflammation of the conjunctiva and sclerotica remain just the same. 8 p.m. The pupil is considerably dilated, and she says that she can see much better; the sclerotic inflammation is also somewhat diminished.—Rep. Ol. Terebinth.

July 5th. Slept quite free from pain last night, but early this morning she was sick and complained of pain in her forehead; these unpleasant symptoms went off after taking some warm tea. The inflammation of the sclerotica is a little increased, but the iris remains the same; her bowels rather confined.

Pulv. Jalap. Comp. ʒi. statim. Rep. Ol. Terebinth.

July 7th. All traces of the inflammation are gone except a very slight tinge on the sclerotica, and she has perfectly recovered her sight; it was, however, thought advisable to continue the medicine for a day longer. The eruption remains exactly the same as when she was admitted. In this case the oil of turpentine did not cause the least pain or difficulty in making water. —Case by Mr. Taylor.

*Case* 2. Sophia Prout, æt. 27, admitted July 13th, 1829. Has had a speck on the cornea for 20 years, in consequence of inflammation. There is slight inflammation of the sclerotica accompanied by shooting pains in the brow, which have continued for three weeks—sight impaired.

Ol. Terebinth. ʒi. ter in die sum.

July 15th. The shooting pains are a little relieved, but the inflammation is much the same. The first doses of turpentine caused a good deal of nausea and vomiting; she continued, however, to take it, and without any unpleasant effects.—Rep. Med.

21st. She has taken the oil of turpentine regularly without suffering any sickness or strangury. The inflammation of the sclerotica is nearly gone, and she does not complain of much pain. —Cont. Med.

28th. There was a slight relapse on the 23d, but she has continued to take the turpentine, and is now quite well, the inflammation having entirely disappeared, and the shooting pains gone. The speck on the cornea of course still remains, and she is to have a drop of the wine of opium in the eye every morning.—Case by Mr. James.

*Case* 3. John Perkins, of Chelsea, æt. 22, a carpenter, was admitted July 21st, 1829, having sclerotic and internal inflammation of the left eye. Says that he was affected with gonorrhœa last November, and a week afterwards applied to a surgeon at Greenwich, who gave him some powders and pills to take, which at first produced vomiting and purging, and ultimately affected his mouth. This treatment was continued about a fortnight, when the running ceased. In February he caught cold, and shortly afterwards observed an eruption over the whole body —for this he took a few powders; the eruption gradually disappeared in the course of two months, with the exception of that on the legs, where a considerable number of scaly blotches, of a copper-colored base may still be seen. In the beginning of May his left eye became inflamed, and he applied to this hospital: he was cupped and the organ shortly afterwards got well again.

The present inflammation has been gradually increasing for the last week; there is a general redness over the sclerotica of a pink colour, and more particularly marked near the cornea, where the injected vessels are very numerous and minute; there is a slight alteration in the appearance of the iris, but the pupil is regular; cornea less bright than usual; complains of deep-seated pain of the eye, pain also on the eye-brow; intolerance of light and indistinct vision.

App. Cucurb. c. Ferro ad Temp. ad ℥xvi. Ol. Terebinth. ʒi. ter die.

July 23d. Has only taken two doses during the day. General vascularity much less, and the pink colour has almost disappeared; pain considerably less, and the light more supportable; sight is more distinct; he has neither experienced any nausea or purging, but the urinary secretion has been increased. —Rep. Med.

25th. Is much better; the redness has almost disappeared; there are merely a few striæ on the upper part of the sclerotica; pain very slight, and only occasional. The medicine appears only to have acted on the kidneys.—Rep. Med.

28th. The inflammation has returned in some degree; light is less supportable, and there is also pain in the eye and forehead.—Rep. Med. ter die.

30th. Considerable amendment; no unpleasant effects from the medicine; tongue clean.—Rep. Med.

August 4th. The inflammation has subsided, and there are now only two or three injected vessels of the conjunctiva to be observed. He says that he feels no inconvenience from the eye, except when very much heated, when it feels rather weak. The blotches on the leg have greatly diminished, and on the right one have nearly disappeared.

7th. Remains well.—Case by Mr. Fisher.

*Case* 4. Ann Dugdale, æt. 26, admitted July 14th, 1829, with syphilitic iritis of the left eye. The inflammation commenced a week ago with very severe pain over her brow, which is worse at night—great intolerance of light. The color of the iris is changed, and the pupil irregular; cornea rather muddy; sclerotica much inflamed.

Cap. Ol. terebinth. ʒj. ter die.

July 15th. The pain over the brow and inflammation remain in the same state as yesterday, and the zone round the cornea is well marked. The turpentine has not acted on the bowels or urinary organs.—Rep. med.

16th. Pain rather relieved; the secretion of urine increased, accompanied by scalding.

Rep. med. Infus. lini. pro potu ordinario.

17th. Much worse in every respect; has taken her medicine regularly, and it has caused much irritation of the bladder and slight purging.

V. S. ad ℥x. ℞. Cal. gr. ij. Opii, gr. ¼. ℳ. ft. pil. ter die sumenda. Illinatur fronti. Ung. hydrarg. ʒss. ter die.

18th. She felt immediately relieved after the bleeding, and the eye looks considerably better.—Rep. pil.

22d. The inflammation is disappearing fast; is quite free from pain; pupil still continues irregular; her mouth rather sore.

Rep. pil. Empl. belladonnæ fronti.

23d. Eye considerably better; mouth very sore.

Omitte pil. Extr. coloc. c. gr. v. h. s. s. Magn. sulph. ℥ss.

23d. The belladonna plaster was repeated last night, and the eye is less inflamed. Rep. pil. et magn. sulph.

24th. The inflammation is now very trifling, but the pupil continues irregular; Gutt. belladonnæ om. man. Her mouth is still very sore. Gargarisma aluminis, sæpe utend.

Aug. 13th. Discharged cured, the eye having resumed its natural appearance. —Case by Mr. C.H. James.

The turpentine failed in this case, and mercury was had recourse to with success.

*Case* 5. Wm. Chalford, æt. 18, admitted July 21st, 1829, having syphilitic iritis of the left eye. Says that he had sores on his penis some time ago. There is now a copper-coloured eruption (lichen syphiliticus) very plentiful on his back and arms, accompanied with nocturnal pains in his shin bones; he has had no sore throat, and has not taken any mercury. His eye has been inflamed the last three days, and he suffers great pain round the orbit, especially at the lower part; his sight is also considerably impaired. The iris is a good deal changed in colour and is immoveable; pupil regular; conjunctiva inflamed, and the vessels of the sclerotica are of a pink colour and very numerous round the margin of the cornea.

Ol. terebinth. ʒj. ter die.

July 22d. Has taken his medicine regularly; makes water more frequently but without any pain; urine is high-coloured. He suffered a good deal of pain in the eye last night, but is now quite free from it; the iris is not so much discoloured, but the redness of the conjunctiva and sclerotica is just the same as yesterday. Says that the medicine makes him feel sick. To continue the turpentine.

23d. The pain round the orbit as bad as ever, but the inflammation is less, and he sees better. Complains of great pain in the glans penis on making water; urine bloody and turbid. To repeat the medicine and drink plentifully of linseed tea.

24th. Pain round the eye not diminished; iris is becoming irregular and more changed in colour, with small globules of lymph adhering to its surface. He has frequent desire to make water; urine bloody, on voiding which he suffers extreme pain in the region of the bladder, extending along the urethra to the glans penis. Ordered to lose ℥x. of blood from his temple, and continue his medicine.

25th. Would not submit to be cupped, and did not take the turpentine yesterday. He sees better, and does not complain of any pain; the redness is not so great. The strangury continues severe. To drink plentifully of linseed tea, and continue the turpentine.

26th. Only took two doses yesterday. The eye remains much the same.

Rep. ol. terebinth. Pulv. jalap. c. ʒj. statim.

28th. Has neglected to take the turpentine since the 25th. He has, however, no pain in his eye, and has quite recovered his sight. The eruption is of a much lighter colour. The belladonna in solution to be dropped into the eye. Pulv. jalap. c. ʒj. The urinary organs continue highly irritated.

30th. The strangury is less severe. The eye continues free from pain, but the redness has not diminished; iris has nearly regained its colour, and is irregular. To commence the turpentine again.

Aug. 8th. He continued the turpentine till the 1st of August, when it was omitted, and the drop of belladonna has since been applied to the eye daily. To-day the eye is more inflamed, but without any pain. Rep. ol. terebinth,

12th. Has continued the medicine regularly up till to-day, and is now nearly well, having no pain, and the redness almost disappeared; iris of its natural colour, and the pupil regular.

13th. Discharged cured.

20th. Returned with a slight degree of redness around the cornea; the lower edge of the iris a little irregular. Apply the belladonna—Pulv. jalap. c. ℈j.

22d. Discharged.

In this case the turpentine arrested the progress of the inflammation several times, and has removed the complaint for the present; the eruption has disappeared, but Mr. Guthrie says he does not think the removal of the iritis or of the eruption will be permanent.—Case by Mr. Foote.

*Case* 6. James Kell, æt. 25, admitted with syphilitic iritis, July 25th, 1829. Complains of great pain over the left eyebrow, accompanied by an itching of the eye, slight intolerance of light, and discharge of tears; his sight too is a little impaired. The conjunctiva and sclerotica are both considerably inflamed; the iris is of a darker colour than natural; pupil dilated, fixed, and regular; cornea quite healthy. The inflammation first appeared a week ago, ushered in with great pain.

℞. Vini rad. colchici, ʒj. Pulv. ipec. comp. gr. x. Aquæ, ʒiss. M. ft. haust. h. s. s.

July 28th. Did not come to the hospital for his draught, and has therefore only taken one dose; the pain is however much relieved, but the redness seems increased.

C. cu. ferro. ad ℥xiv. Pulv. jalap. comp.

30th. The inflammation is a good deal diminished, and he is in no pain; the iris is in the same state as when he was admitted.

Haust. colchici c. opio, h. s. s.

31st. Much worse to-day; inflammation of the sclerotica considerably increased; iris irregular, and the pains over the brow returned. He says that he had a chancre last Christmas, which was cured by mercury, and that nothing more of the venereal disease showed itself till seven weeks back, when an eruption (lichen syphiliticus) appeared on his arms and legs, where it is even now visible, and very characteristic.

Capiat ol. terebinth. ʒj. ter die. Pulv. jalap. c. ℈j. statim.

Aug. 1st. The powder acted well on his bowels before he took any of the turpentine; after the second dose, however, he experienced great pain in making water, and since the third dose it has been almost unbearable. The inflammation is rather worse, but the pains over the brow are relieved. To take the turpentine only twice a day, and to drink plentifully of linseed tea.

Sulph. mag. ℥j. statim.

2d. Says that his sight is improved, but the pains continue very severe over his brow. Only took one dose of turpentine yesterday, as the strangury was so very bad.—Rep. ol. tereb. bis die. Pulv. jalap. c. ℈j.

3d. The difficulty in making water, attended with pain and dripping, still continues. The inflammation of the sclerotica is a good deal diminished, but the pupil continues very irregular; the pain over the brow quite gone.

Rep. ol. terebinth. Infus lini. cu. potass. nitrate sæpe.

4th. Much the same as yesterday—only took one dose of turpentine.—Rep. medicamenta.

5th. Not so well; some degree of pain over the brow with increased lachrymation. The ol. tereb. gives him so much pain that he is to omit it to-day, and to take a purgative.

7th. Took three doses of the turpentine yesterday, but has since passed blood after making water. The inflammation has very much diminished; the iris acts, and the pupil is more circular.

Omitte ol. terebinth. Pulv. jalap. comp. ℈j. statim.

11th. Has taken the turpentine every other day, although suffering a good deal of pain and difficulty in making water. The redness of the sclerotica has almost disappeared, and he has not suffered any pain in the eye or over his brow for the last two days; the pupil is quite circular, but the iris is rather sluggish in its motions.—Rep. med.

13th. Nearly well.

18th. Discharged cured.—Case kept by Mr. Taylor.

